# Bias in recent miRBase annotations potentially associated with RNA quality issues

**DOI:** 10.1038/s41598-017-05070-0

**Published:** 2017-07-12

**Authors:** Nicole Ludwig, Meike Becker, Timo Schumann, Timo Speer, Tobias Fehlmann, Andreas Keller, Eckart Meese

**Affiliations:** 1 Department of Human Genetics, Saarland University, Homburg, Germany; 2 Department of Internal Medicine, Nephrology and Hypertension, Saarland University Medical Center, Homburg, Germany; 3 Chair for Clinical Bioinformatics, Saarland University, Saarbrücken, Germany

## Abstract

Although microRNAs are supposed to be stable *in-vivo*, degradation processes potentially blur our knowledge on the small oligonucleotides. We set to quantify the effect of degradation on microRNAs in mouse to identify causes for distorted microRNAs patterns. In liver, we found 298, 99 and 8 microRNAs whose expression significantly correlated to RNA integrity, storage time at room temperature and storage time at 4 °C, respectively. Expression levels of 226 microRNAs significantly differed between liver samples with high RNA integrity compared to liver samples with low RNA integrity by more than two-fold. Especially the 157 microRNAs with increased expression in tissue samples with low RNA integrity were most recently added to miRBase. Testing potentially confounding sources, e.g. *in-vitro* degraded RNA depleted of small RNAs, we detected signals for 350 microRNAs, suggesting cross-hybridization of fragmented RNAs. Therefore, we conclude that especially microRNAs added in the latest miRBase versions might be artefacts due to RNA degradation. The results facilitate differentiation between degradation-resilient microRNAs, degradation-sensitive microRNAs, and likely erroneously annotated microRNAs. The latter were largely identified by NGS but not experimentally validated and can severely bias microRNA biomarker research and impact the value of microRNAs as diagnostic, prognostic or therapeutic tools.

## Introduction

MicroRNAs (miRNAs) are small non coding RNAs, that can inhibit translation of genes in a sequence dependent manner^1^. To generate a common database on miRNA sequences, the miRBase registry was created in 2002, starting with 218 entries^2^. Currently in its 21^st^ version, the miRBase contains 35828 mature miRNA sequences from 223 species, including 2588 and 1915 mature miRNAs for human and mouse, respectively^2, 3^. Initially, miRNAs were identified through laborious Sanger sequencing of cDNA clones from small RNA libraries and only a few tens to hundred novel miRNAs were added to the database in each novel version. But with the advent of next generation sequencing, this number dramatically increased to thousands^4^. While many of the miRNAs from the early versions of miRBase have been experimentally validated by Northern blotting, such evidence is lacking for most miRNAs introduced by NGS.

In contrast to mRNAs, miRNAs show higher stability and lower susceptibility for degradation^5–8^. This might be in part due to their short length of only 18–22 nucleotides, but also because of their association with proteins like AGO^9^. Determination of half-lives of miRNAs in living cells showed heterogeneous results, with some miRNAs displaying half-lives of over 24 hours, while others reaching half-lives of only 4–14 hours^10^. Interestingly, passenger strand miRNAs were preferably found in the group of miRNAs with short half-lives. Aside from the situation in living cells, there is a debate about stability of miRNAs in solutions, e.g. serum or plasma, or in degrading tissues samples and their usability in profiling studies^7, 11, 12^. It is, however, not clear what level of RNA degradation is still acceptable for performing miRNA profiling. Recommendations for an acceptable RNA integrity range from a RIN of 3.95 to 8 depending on the analysis method^12–15^. The few studies interrogating the stability of miRNAs in degraded RNA samples mostly examined only small numbers of miRNAs. They are also difficult to compare because of their heterogeneity: they use different models of RNA degradation, tissues and methods^5, 6, 8, 12, 15–17^. MiRNA degradation seems to vary in different tissues, most likely dependent on levels of RNases^6, 12, 16, 17^. *In vitro*, miRNAs are not sensitive to heat treatment, but application of UV, RNase A, RNase I_f_ and NaOH seems to degrade even miRNAs^5, 8, 15^. Only two studies examined profiles of extended numbers of miRNAs by using microarrays. Both found an impact on the expression profile with miRNAs both with increased and decreased expression^12, 18^.

The aim of our study is to further our knowledge about the stability of individual miRNAs and miRNA expression profiles in different tissues under the influence of degradation processes. We examined the miRNome of mouse heart, liver and brain tissue samples with different cold-ischemic storage times and temperatures. Besides miRNAs that showed low abundances in highly degraded tissues samples, there were miRNAs with elevated abundances in degraded tissues samples. By carrying out systematic control experiments we provide evidence that the latter miRNAs likely result from RNA degradation processes and are erroneously termed miRNAs. Notably, these “false” miRNAs have mostly been deposited in the more recent versions of miRBase and have been identified by NGS without further experimental validation.

## Results

### RNA integrity during tissue degeneration

**Figure 1:**
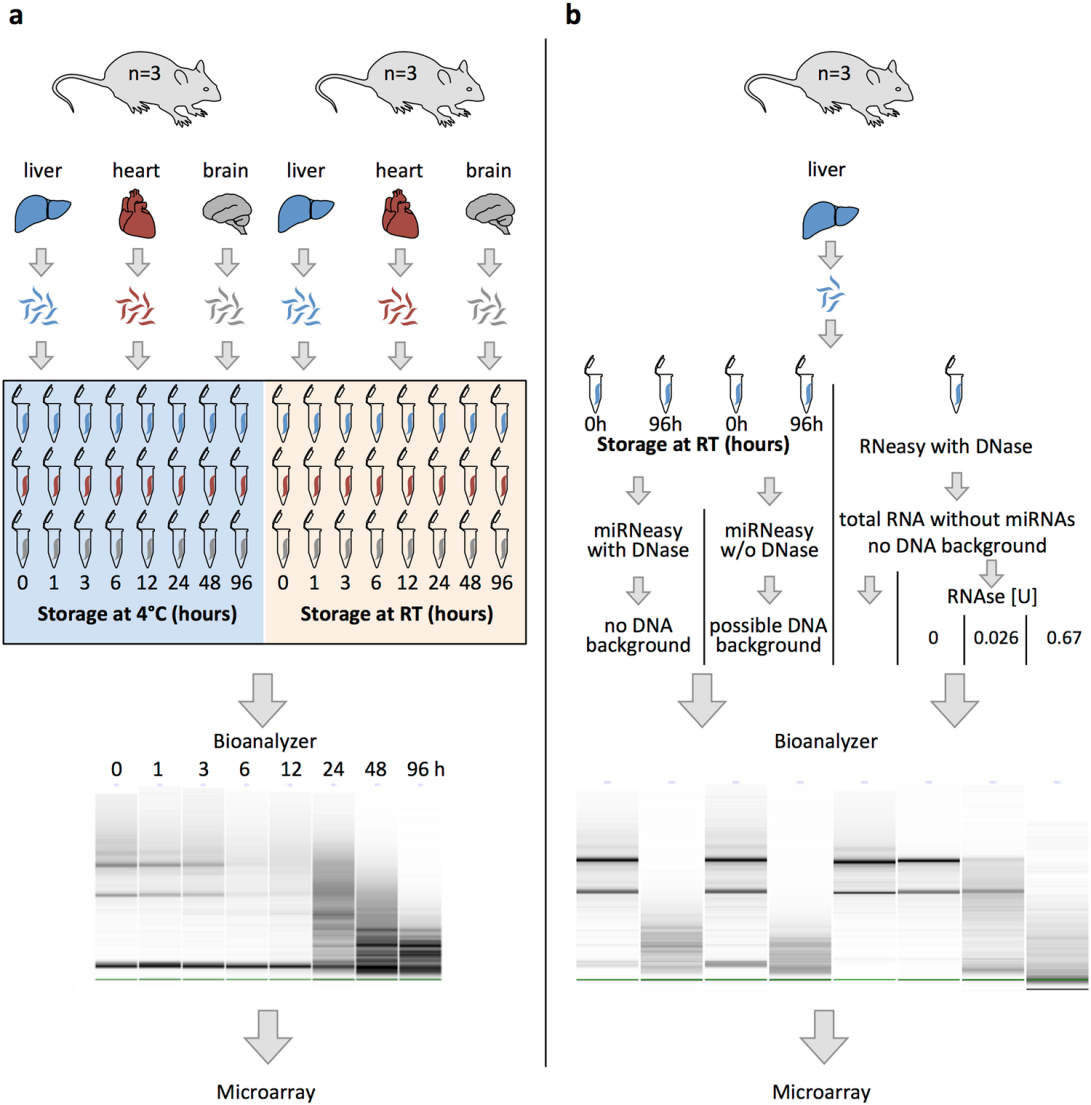
Experimental design. (**a**) Liver, heart and brain of male mice were harvested immediately after death, divided into 8 parts of about equal size, and stored at either 4 °C or at room temperature (RT) for the indicated time periods before RNA isolation. Experiments were performed in biological triplicates. RNA integrity was measured with Bioanalyzer. Gel-like image of brain tissue is given as example. MiRNA expression profiles of one replicate were measured using microarrays. (**b**) Liver tissue of 3 male mice was harvested immediately after death and divided into 5 parts of about equal size. Three parts were immediately transferred into RNAlater (0 h), two parts were stored for 96 h at room temperature (96 h). Two samples (0 h and 96 h) were isolated using standard procedure with miRNeasy Kit without DNase digestion. Two samples (0 h and 96 h) were isolated with optional DNase digestion to exclude DNA background. From the remaining undegraded sample (0 h), total RNA without small RNAs was isolated using RNeasy Kit with optional DNase digestion. Isolated RNA was further treated with 0 U, 0.026 U and 0.67 U RNase for 30 min to generate artificial RNA degradation. RNA integrity was measured with Bioanalyzer. MiRNA expression profiles of all replicates were measured using microarrays. The schematic drawings were prepared using the Biomedical-PPT-Toolkit-Suite from Motifolio Inc., USA.

We isolated RNA of heart, brain and liver mouse tissues that were stored for 0–96 hours at 4 °C and RT, respectively, and analysed RNA integrity using Bioanalyzer (Fig. 1a). As measure of RNA integrity, we applied the RIN value, which spans from 10 indicating fully intact RNA to 1 indicating severely degraded RNA. Representative gel-like images of RNAs for all tissues and the mean RIN values obtained from three biological replicates for the different time points, tissues and temperatures can be found in Supplementary Fig. S1 (see also Supplementary Table S1 online for individual RIN values of all samples). As expected, all tissues degraded faster at RT than at 4 °C, with liver being the tissue with highest degradation rate at both temperatures. At 4 °C, degradation occurs in a linear rate in all tissues, with brain and heart showing the least degradation at 4 °C (RIN values >7 even after 96 hours). At RT, degradation seems to be tissue specific with RIN values in brain declining in a linear fashion throughout the time course. RIN values in heart showed a two-phase decrease with a plateau phase during the first 24 hours without decrease followed by linear decrease until 96 hours. In contrast, degradation in liver samples shows a hyperbolic course, with RIN values dropping to a level of 4 after only 6 hours of storage. The high standard deviation in the biological replicates indicates the influence of additional factors like inter-individual differences or tissue sample size on the level of degradation. While these results show that RNA degradation is tissue and temperature dependent, the results also indicate other yet unknown factors that can interfere with RNA degradation, which is in line with previous observations by others^12, 19, 20^.

### Influence of degradation on miRNA profiles

**Figure 2:**
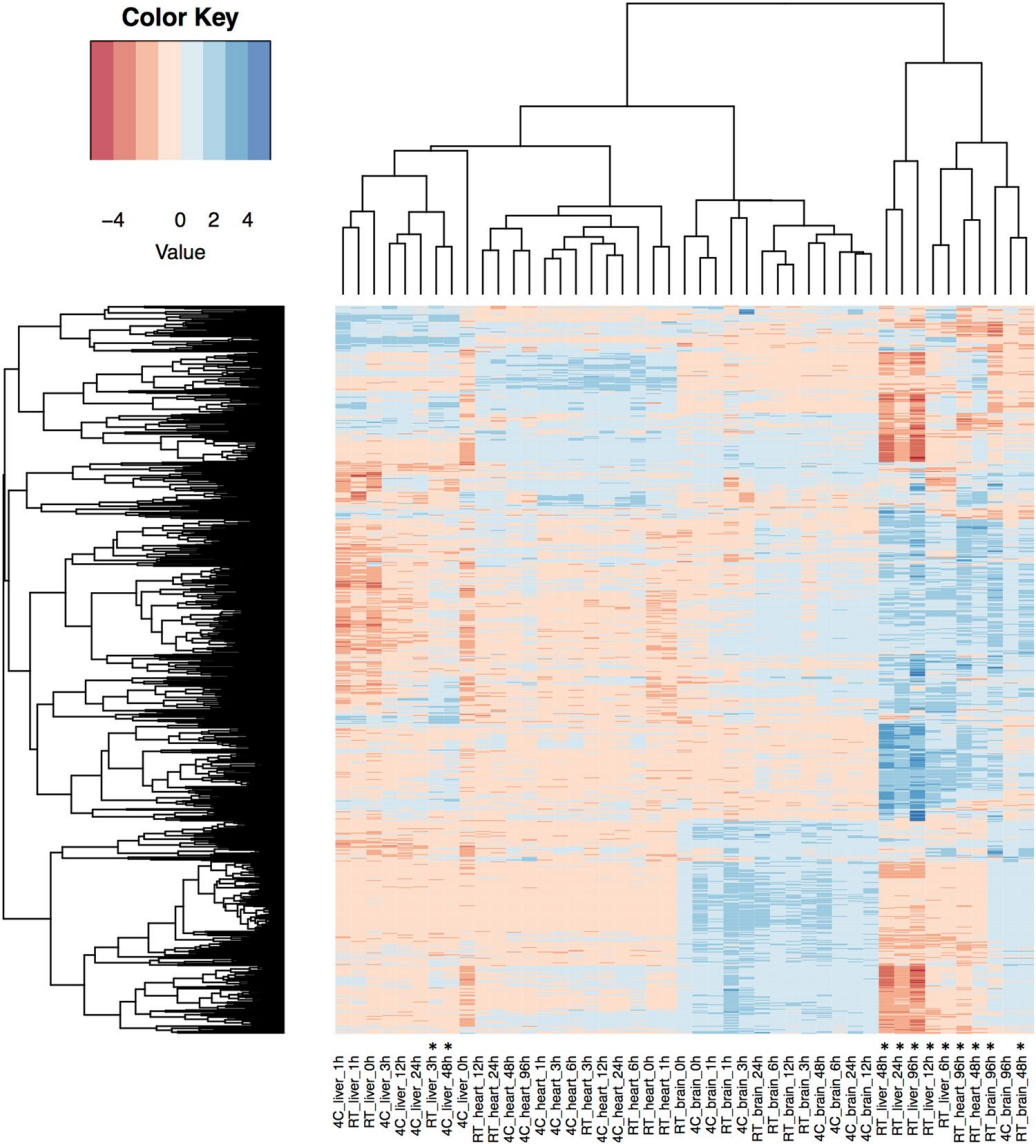
Influence of degradation on miRNA expression profiles as determined by microarrays. Unsupervised hierarchical clustering using Eucledian distance and complete linkage of the samples depending on expression all analysed miRNAs. Blue color indicates high, red color indicates low expression. Samples are indicated on the X axis below the heatmap with each sample referring to the storing temperature, the tissue and the storage time in hours (e.g. 4C_heart_24 h denoting the heart sample that was stored for 24 hours at 4 °C). Samples with RIN < 6 are marked with an asterisk. Clustering of the samples is shown above the heatmap. MiRNA clusters are indicated on the left-hand side of the heatmap.

To determine the influence of degradation on miRNA profiles, we analysed the mouse miRNome by microarrays. We studied the expression of 1,881 mouse miRNAs (miRBase version 21) of the 3 tissues at 8 different time points at two different temperatures totalling 48 analyses. Three samples were removed from the analysis due to low quality microarray data, i.e. liver 6 and 96 hours at 4 °C and heart 0 hours at 4 °C. Furthermore, all miRNAs detected in less than five samples were excluded, leaving 815 miRNAs for further analysis. Unsupervised hierarchical clustering based on the 50 miRNAs with highest variance showed perfect separation between the three tissues (Supplementary Fig. S2). Clustering based on all 815 miRNAs, however, showed that severely degraded samples fell in one group irrespective of tissue of origin with the exception of two liver samples that were stored for 3 h at RT and for 48 h at 4 °C (RIN of 4.1, each) clustering with the non-degraded liver samples (Fig. 2). Samples with higher RNA integrity are separated into three tissue-specific clusters with the exceptions of a brain sample that was stored for 96 h at 4 °C (RIN of 7.3) clustering with the other degraded tissues. These results are in contrast with the previous study of Ibberson *et al*. that reported tissue-specificity of miRNA profiles even in severely degraded RNA samples ^12^. This difference can, however, be explained by the longer time of tissue degeneration and the higher number of miRNAs examined in our study as compared to the study of Ibberson, who analysed expression of 500 mouse miRNAs (miRBase v9.2) in samples stored up to 4 hours after tissue removal.

### Correlation of miRNA expression to RNA integrity value and storage time

To determine the effects of RNA degradation on expression levels of individual miRNAs in the three tissues, we computed the Pearson correlation coefficient for each miRNA correlating its expression value with the RIN integrity number of the sample and with the storage time at either 4 °C or RT. Of the 815 analysed miRNAs, the expression level of 298 miRNAs correlated significantly with RIN value (Supplementary Table S2) with only 84 showing a positive correlation (>0.5) between expression and RIN values. The remaining 214 miRNAs showed a strongly negative correlation (<−0.5) of their expression to RIN values, i.e. a high abundance in samples of low quality. As for the storage time, we found 8 miRNAs showing a significant correlation between miRNA expression and the storage time at 4 °C and 99 miRNAs with a significant correlation between expression and the storage time at RT. At 4 °C, only miR-652-5p showed a lower expression in the samples with extended storage, while the remaining seven miRNAs showed an elevated expression in the samples with extended storage. At RT, 15 miRNAs showed a lower expression in the samples with extended storage and 84 miRNAs an elevated expression in the samples with extended storage. Expression levels of the miRNAs with highest positive and negative correlation to RIN values and storage times are given in Supplementary Fig. S3.

**Figure 3:**
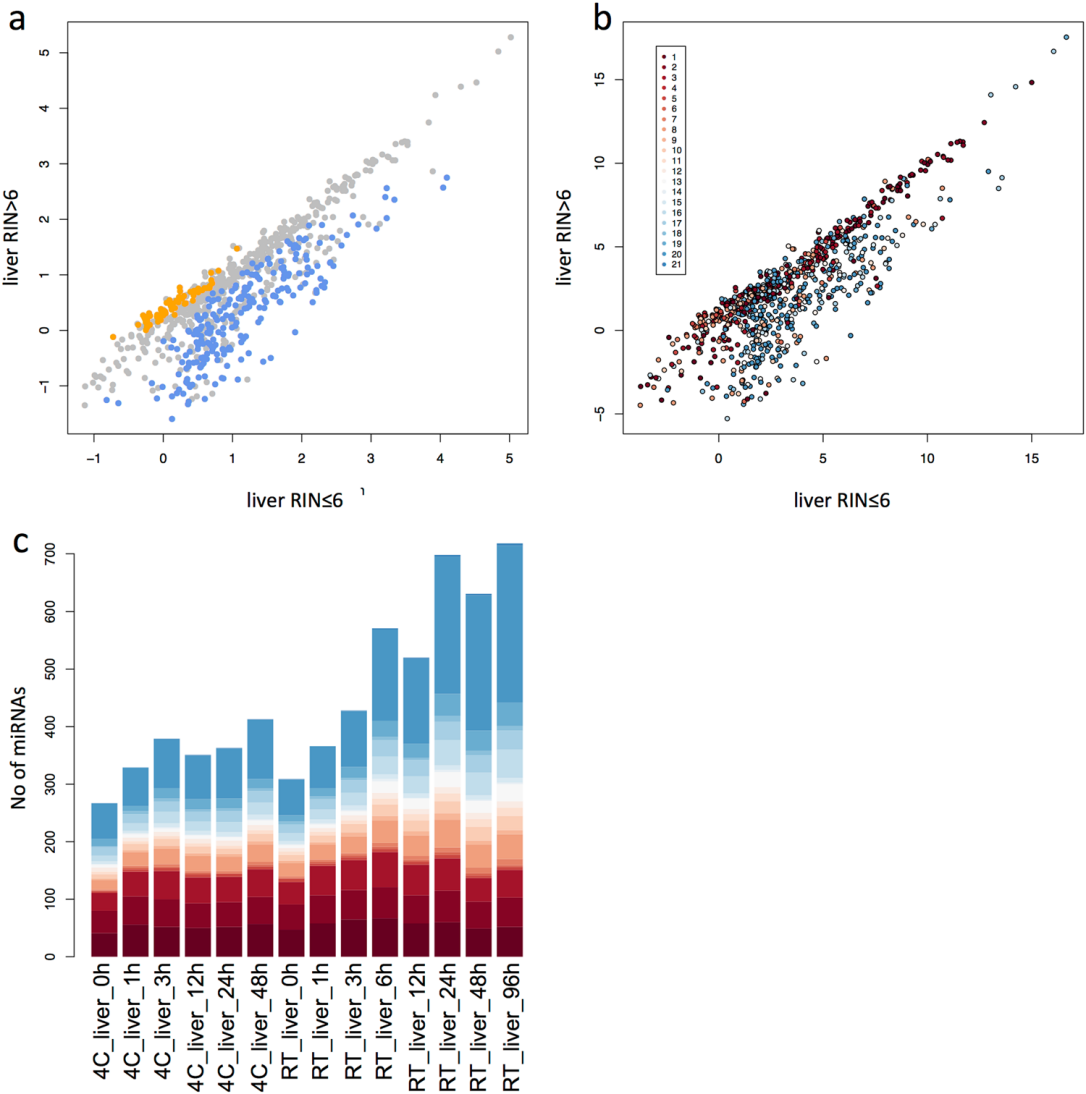
MiRNA expression in liver samples with low RNA quality as compared to their expression in samples of high quality. (**a**) Median expression of miRNAs in liver samples with low RNA integrity (RIN ≤ 6) are indicated on the X- axis and median expression of miRNAs with high RNA integrity (RIN > 6) on the Y-axis. Each point represents a miRNA with orange points indicating miRNAs that are significantly lower expressed in samples with low RNA integrity and with blue points indicating miRNAs that are significantly higher expressed in samples with low RNA integrity. (**b**) The same blot as in Fig. 3a, but with miRNAs color-coded according to the miRBase version, in which the miRNAs were first described. (**c**) Total number of miRNAs detected in the liver samples during the time-course experiment according to the miRBase version, in which the miRNAs were first described. Color-codes for the miRBase version as in Fig. 3b.

Since RNA degradation was more severe in liver samples than in brain or heart, we further analysed the liver samples for miRNAs significantly affected by degradation. We found 226 differentially expressed miRNAs in liver samples with a RIN > 6 (high quality) versus liver samples with a RIN < 6 (low quality) (WMW test, p < 0.05). Out of these 226 miRNAs, 157 showed more than two-fold increased levels in samples with low quality, while 17 showed more than two-fold decreased levels (Fig. 3a). Notably, most of the miRNAs with a higher expression in the low-quality samples were deposited in miRBase versions >16 (Fig. 3b). As this was intriguing, we analysed the total number of detected miRNAs in these samples. The total number of miRNAs increased from 267 miRNAs at 0 h to 413 miRNAs at 96 h when the samples were stored at 4 °C, and from 309 to 718 miRNAs when the samples were stored at RT. As seen in Fig. 3c, this dramatic increase in detected miRNAs at RT is mainly due to miRNAs that were added in miRBase versions 16–21 (108 miRNAs at 0 h vs 407 miRNAs at 96 h), whereas the number of detected miRNAs from the first miRBase versions only increased slightly (130 miRNAs from miRBase versions 1–3 at 0 h to 151 miRNAs at 96 h). From these analyses we conclude that, although we find degradation of some well known miRNAs during total RNA degradation, we also find a substantial number of newly detected miRNAs in the degraded samples. In addition, most of these miRNAs with increased expression in the degraded samples have been added to the miRBase in their latest versions, leading to the question, if these miRNAs are “true” miRNAs.

### Identification of false positive miRNAs

**Figure 4:**
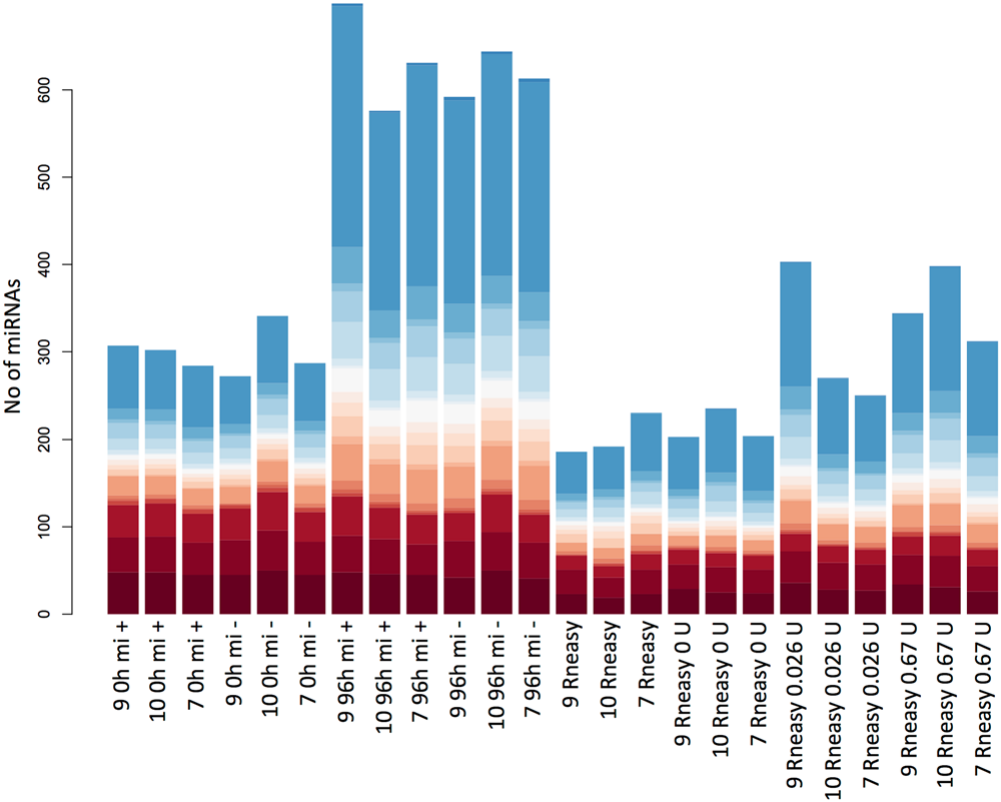
Number of detected miRNAs in the samples according to miRBase version. Total number of detected miRNAs in the replicate samples. The coloring indicates the proportions of miRNAs from miRBase version 1 (red) to 21 (blue) that were detected in the respective samples. Color codes are identical to Fig. 3b and c.

To further clarify the nature of the more recently deposited miRNAs that show an elevated expression in the low-quality samples, we performed additional degradation experiments in liver samples and analysed total miRNA expression by microarray. As potential confounding source for the hybridization signals on the array we assumed either fragmented RNAs or even fragmented DNAs. Therefore, we included total RNA that was depleted of small RNA molecules and degraded by different concentrations of RNase as source of fragmented RNAs (Fig. 1b). Furthermore, we used fresh liver tissue and liver tissue stored for 96 h at RT each with and without DNase digestion, as potential source of contamination with fragmented DNA. For the sake of simplicity, we refer to the hybridization signals in the following as miRNAs, being aware that some experiments were performed with total RNA largely depleted of small RNAs. We obtained hybridization signals in all experiments including the experiments with the RNA that was depleted of miRNAs. In detail, in the samples with intact RNA including small RNAs we detected expression of about 300 miRNAs, while in the respective samples with degraded RNAs, the number of detected miRNAs increased to over 600 (Fig. 4). Moreover, in the samples with intact RNA depleted of small RNAs we still detected expression of about 200 miRNAs, while in the respective samples with partially or fully degraded RNA, the number of detected miRNAs increased to 300 and 350, respectively. Interestingly, while the number of miRNAs from miRBase versions 1–3 remained fairly constant in degraded compared to non-degraded RNA samples (mean 122 miRNAs for intact RNA and 124 for degraded RNA), the number of miRNAs from miRBase versions 16–21 increased substantially (115 vs 372).

**Figure 5:**
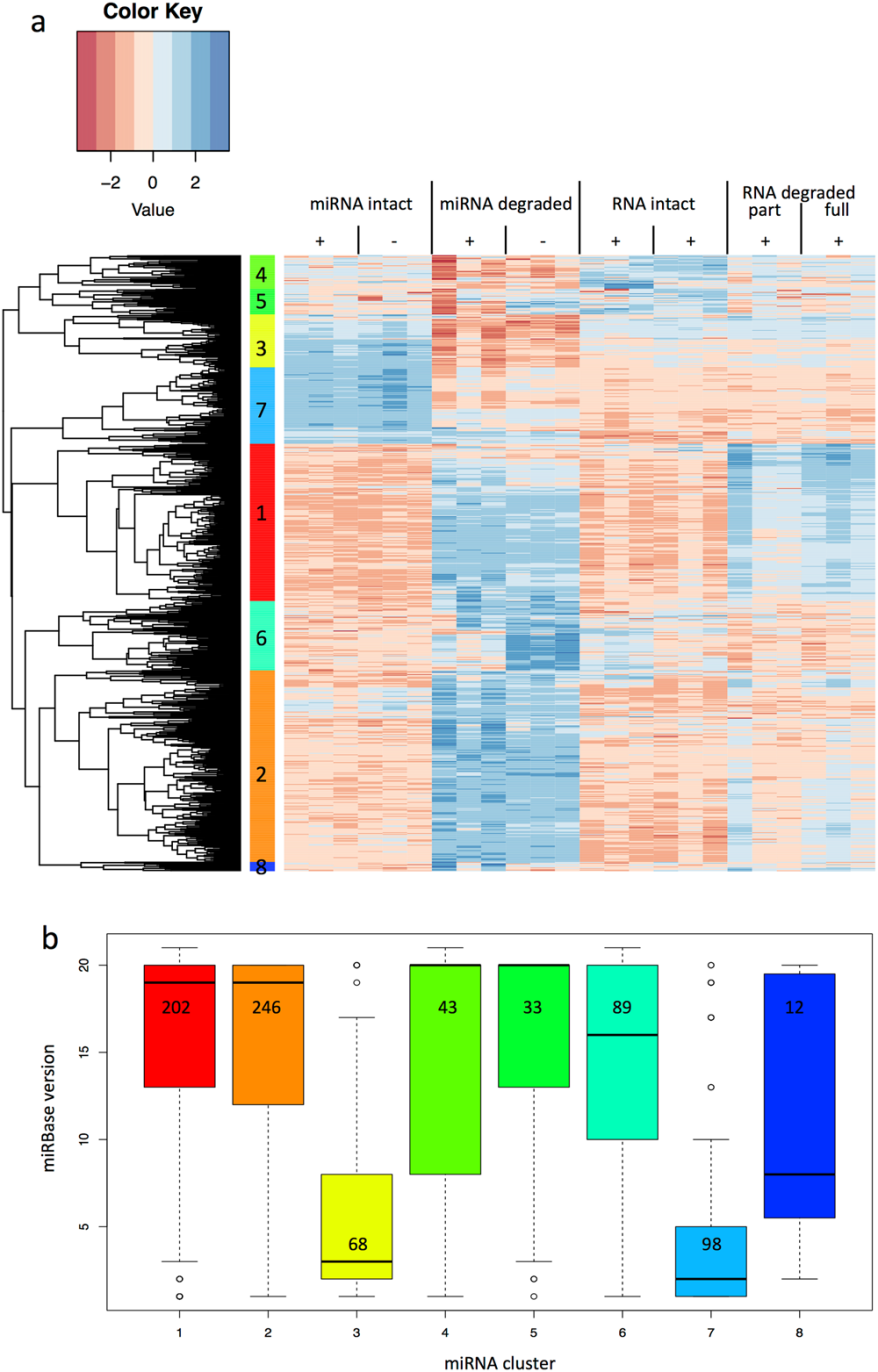
Influence of degradation and RNA isolation methods on miRNA detection using microarrays. (**a**) Unsupervised hierarchical clustering of miRNAs using Eucledian distance and complete linkage in the samples.The treatment and the source of RNA are indicated above the heatmap (see Fig. 1). “miRNA intact” stands for RNA including small RNA isolated from fresh liver tissue, “miRNA degraded” for RNA including small RNA from liver tissue stored for 96 h at RT, “RNA intact” for RNA depleted of small RNA from fresh tissue, and “RNA degraded” for RNA depleted of small RNA molecules and degraded by different concentrations of RNase. “+/−” indicating presence or absence of DNase digestion during RNA isolation. The expression of miRNAs in the heatmap are color coded with blue indicating high and red low expression. MiRNAs cluster according to their expression in eight clusters marked with colored boxes and numbers 1 to 8 on the left side of the heatmap. A list of the miRNAs belonging to each cluster is given in Supplementary Table S3. (**b**) Relationship between miRBase deposition of miRNAs and their clustering according to degradation. The eight miRNA cluster as determined in Fig. 4a are listed on the X-axis with the same color code as above. The box-plot shows miRBase version, in which the miRNAs in the respective clusters were first annotated. The numbers of miRNAs in each cluster are given in the respective boxes. The miRBase version is given on the Y-axis (i.e. 1-21).

**Figure 6:**
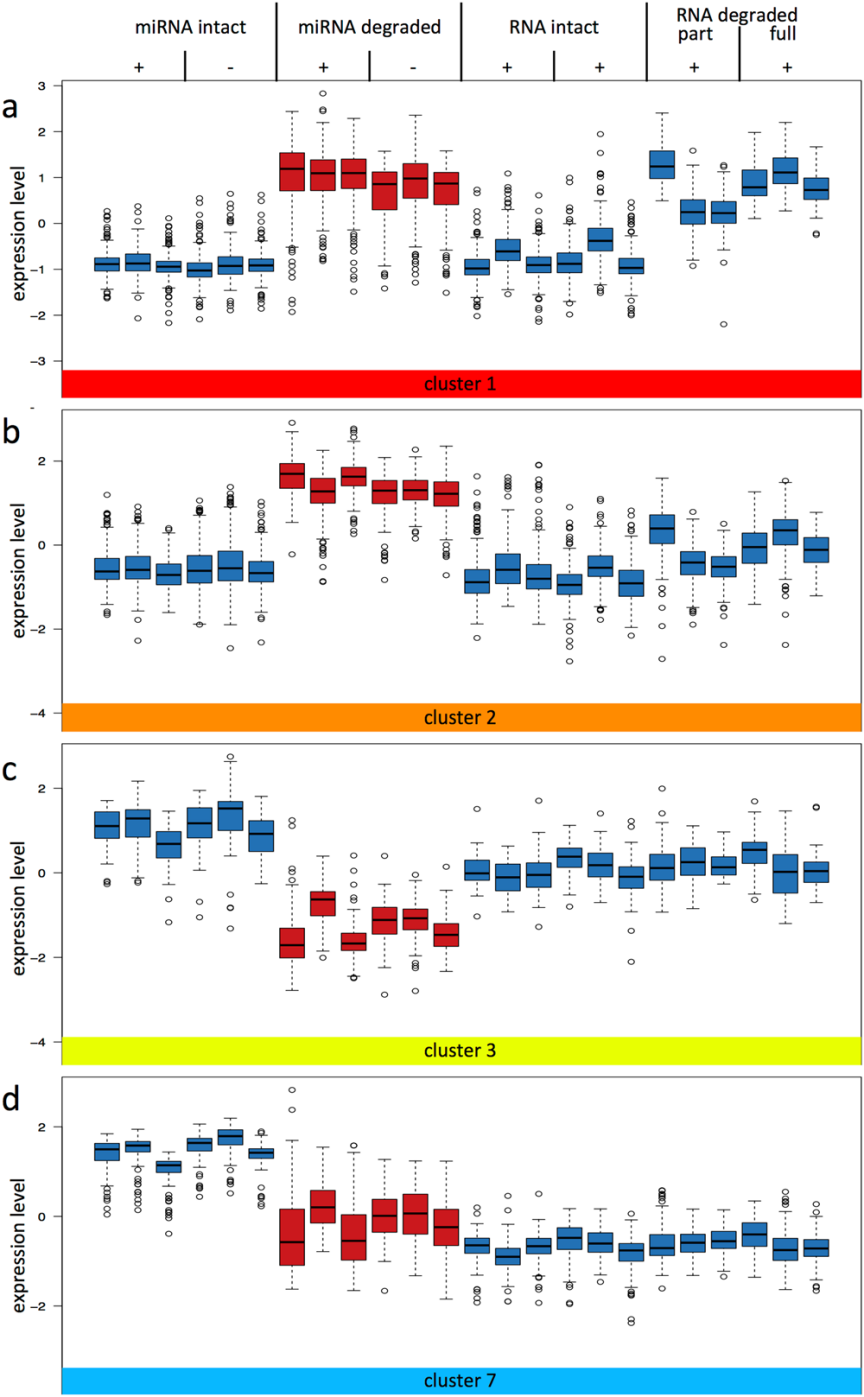
Expression of miRNAs in selected miRNA clusters. Box-plots of the median expression of miRNAs (Y axis) in liver tissues are shown for treatment/source of RNA as indicated above the box-plots (description is as in Fig. 5). The selected cluster is indicated by the same color code and number as in Fig. 5 as shown below the box-plots. Figure 6a shows cluster 1, 6b cluster 2, 6c cluster 3 and 6d cluster 7. Respective box-plots for the remaining clusters can be found in Supplementary Fig. S4. The list of miRNAs belonging to each cluster can be found in Supplementary Table S3.

Unsupervised hierarchical clustering of the hybridization signals on the microarray identified eight different clusters (Fig. 5a, Supplementary Table S3). The majority of the 791 analysed miRNAs were found in clusters 1 and 2 that contain 202 and 248 miRNAs, respectively (Fig. 5b). Both cluster 1 and 2 show an overall low expression of miRNAs from fresh liver tissues and an elevated expression of miRNAs in liver tissues stored for 96 h at RT. The first cluster contained also elevated miRNA signals obtained when partially or fully degraded total RNA depleted of small RNAs was used as hybridization probe indicating that most hybridization signals of the first cluster were due to sequence homology of RNA fragments to the oligonucleotide probes on the array (Fig. 6a). As for the second cluster, we found only elevated expression in liver tissues stored for 96 h at RT but no comparable increase of the expression level for any of the other sources i.e. total RNA depleted of small RNA either degraded or intact (Fig. 6b). A possible explanation for the increase in signal intensity would be the detection of fragmented RNAs with sequence homology to the miRNAs in these clusters. As the protocol for RNA labelling of the hybridization probes include undirected 3´-labelling, all RNA species in the input RNA would be labelled. In case of a degraded input RNA, not only miRNAs, but also fragments of mRNAs or other RNA species would be labelled. If these fragments share sequence homology with miRNAs, the labelled fragments can bind to the microarray capture probe and can subsequently be detected as a hybridization signal. The difference of signals between clusters 1 and 2 in the degraded RNA samples with depleted small RNAs may result from a difference in sequence homology of the miRNA with other RNA species, i.e. miRNA in cluster 2 might show a lesser level of sequence homology to other RNAs than the miRNAs in cluster 1.

Clusters 3 and 7 contain 68 and 98 miRNAs, respectively, and show a markedly reduced overall miRNA expression in liver tissue stored for 96 h at RT as compared to fresh liver tissue (Fig. 6c and d). This is indicative that these miRNAs are degraded during the tissue decay. Both clusters 3 and 7 do not show a major difference in the overall expression between the RNA, which was miRNA depleted and degraded, and the RNA, which was miRNA depleted but from fresh tissue. Clusters 3 and 7 differ, however, in that the overall expression level for the depleted RNA in cluster 7 was lower than the overall expression level for miRNAs of liver tissue stored for 96 h at RT. For both cluster 3 and 7 there seems to be a limited impact of the overall total RNA degradation on the miRNA pattern of liver tissue stored for 96 h at RT. Box-plots of the remaining clusters can be found in Supplementary Fig. S4, a detailed list of the miRNA belonging to the respective clusters is provided in Supplementary Table S3.

Based on the abovementioned link between the quality of the samples and the time point at which the miRNA was deposited in miRBase, we related the 8 clusters to the different miRBase versions (Fig. 5b). The first cluster with the elevated miRNA hybridization signals that were largely due to the degraded total RNA, contained mostly miRNAs that were deposited in miRBase versions >13. Since these signals were likely due to sequence homology of RNA fragments to the oligonucleotide probes on the array, the according miRNAs in miRBase are potentially erroneously termed miRNAs. Likewise, the miRNAs that fell in cluster 2 were also mostly deposited in miRBase versions >13. Out of the 8 clusters as depicted in Fig. 5b only the clusters 3 and 7 contained miRNAs that were deposited mainly in miRBase versions <10. Since the miRNAs of these two clusters showed the expected reduced expression as result of the tissue degradation, it is likely that they represent true miRNAs and that the early miRBase versions mostly contain “true” miRNAs as compared to the most recent miRBase versions. Moreover, analysis of sequence homology of the miRNAs contained in the different clusters to mRNAs revealed that about 15% of miRNAs in cluster 3 and 7 show an overlap of minimum 90% with a mouse mRNA (with one nucleotide mismatch allowed) whereas over 30% of miRNAs in clusters 1 and 2 show such a homology (Supplementary Fig. S5). It is likely that mRNA fragments strongly interfere with detection of the miRNAs belonging to clusters 1 and 2 in the microarray system. However, in contrast to our assumption above, the miRNAs in cluster 2 showed a higher level of sequence homology to other RNAs than the miRNAs in cluster 1, indicating that sequence homology might not be the only feature influencing signal detection.

DNase digestion influences miRNA detection levels, but only in the degraded samples. In detail, the 89 miRNAs in cluster 6 (see Supplementary Fig. S4) show an overall low expression in the intact RNA samples with and without small RNAs irrespective of the DNase digestion. In the degraded samples including small RNAs, the expression values were markedly elevated in case the DNase digestion step was omitted. Apparently, DNA fragments appear to interfere with detection of these 89 miRNAs in the microarray system.

## Discussion

Since the availability of clinical samples, especially for rare diseases, is often limited with respect to number and/or size of samples, it is sometimes unavoidable to use tissue samples of compromised quality to achieve sample numbers high enough to perform reliable statistical tests for the clinical question considered. In case of gene expression studies, it is common knowledge that RNA quality significantly influences the expression profile as result of tissue degeneration. Since mRNAs are highly susceptible to degradation through RNases, their expression/detection levels can be altered substantially even with low level degradation^21^. However, there is some dispute about what level of RNA degradation is still acceptable for performing miRNA profiling experiments with either sequencing, microarray or qPCR methods. Recommendations for an acceptable RNA integrity range from a RIN of 3.95 to >8 depending on the analysis method^12–15^. For analysing miRNA expression profiles, this issue is even less clear. While several studies claim that miRNAs have high stability even in degraded RNA, other studies show the opposite^5, 8, 12, 15^. In isolated RNA, miRNAs can be degraded by UV, RNase A, RNase I_f_, and NaOH, whereas they are resistant against degradation by heat, RNase H, benzonase and exonucleases T and T7^5, 8, 15^. When storing rat cadavers at different temperatures and post-mortem intervals, stability of miRNAs exceeded those of mRNAs but was dependent on temperature and tissue analysed^6, 16, 17, 20^. However, only a few miRNAs were analysed in each study using RT-qPCR based approaches. In contrast, when analysing miRNA expression profiles using microarrays, two studies found a profound influence of RNA integrity on miRNA expression profile, with some miRNAs showing decreased and a high number of miRNAs showing increased expression levels in degraded samples^12, 18^. Our results point into the same direction. We found that RNA quality is dependent on temperature, tissue and storage time. The miRNA expression profiles of tissues stored at RT for extended periods of time showed marked deviations from the expression patterns of fresh frozen tissues, including miRNAs with decreased but also miRNAs with increased expression in degraded samples. In the light of these findings, it is important to be aware of and to control for pre-analytical factors, *in vivo* as well as *in vitro*, influencing the sample and to properly document these factors in prospectively collected specimens for bio banking, e.g. following the guidelines presented by Khan *et al*. or in the “Sample PREanalytical code” (SPREC)^22, 23^. Aside from the *in vitro* pre-analytical factors described above, also day-time of sample collection can be a potential bias. Several studies have shown that miRNAs can exhibit diurnal expression variation in tissues^24, 25^ as well as in circulation^26, 27^. To minimize a potential bias by these factors in a biomarker discovery study, we propose to use only samples with high RNA integrity, e.g. RIN > 8. However, in subsequent validation studies of a proposed biomarker, it might be feasible to also include samples with lower quality or samples potentially affected by pre-analytical factors as detailed above, to see whether RNA quality or any of these factors affect the biomarker levels.

As second major result we found evidence that miRNAs identified in the later versions of miRBase tend to show an increase in expression in degenerating tissues. We hypothesize that these miRNAs are not “true” miRNAs, but degradation fragments of other RNAs. To test this hypothesis, we isolated RNA largely depleted of miRNAs, degraded the RNA with RNases *in vitro* and analysed the expression profile in comparison to undegraded RNA depleted of miRNAs and in comparison to samples with degraded and undegraded RNA both of which not depleted of miRNAs. Many of the miRNAs that showed increased expression in decaying tissue, also showed high levels of expression in the degraded RNA samples that were depleted of miRNAs. There was, however, no expression of the miRNAs with high expression in decaying tissue, in the undegraded RNA samples independent whether they were depleted or not for miRNAs. This confirmed our hypothesis, that many of the newly discovered miRNAs might indeed be not “true” miRNAs but fragments of other RNAs, in due consideration that other experimental factors might influence these results.

Originally, miRNAs were defined as class of small RNAs of about 22 nt length, that are typically transcribed by RNA polymerase II and produced by specific cleavage of an about 60-80 nt long precursor RNA. This precursor RNA forms a characteristic hairpin structure and is sequentially cleaved by the enzymes DROSHA and DICER, producing two mature miRNAs (-3p and -5p) with characteristic 2 nt overhang at the 3′end^1^. The two small RNAs are located on opposite sides of the hairpin with considerable sequence complementarity, but only one of the short RNAs, either the -3p or the -5p form, is incorporated into the Ago complex and therefore sufficiently stable to infer a biological effect^1^. A common database, the miRBase registry, has been created in 2002, to unify the definition and nomenclature of miRNAs and to provide access to sequences and other information on already identified miRNAs^28^. For a novel miRNA to be included in the database, this miRNA has to meet the following criteria: Presence of a 22nt long sequence detected with either Northern Blot or in a small RNA cDNA library (perfectly matching a genomic sequence of the respective organism) represent strong and weak expression evidence, respectively. Biogenesis criteria include i) the prediction of a hairpin structure containing most of the 22nt sequence within one arm (along with some additional structural requirements), ii) phylogenetic conservation of the sequence and the predicted hairpin structure, and iii) accumulation of the predicted precursor sequence in cells with reduced DICER activity^28^. However, with the identification of more and more miRNAs, also exceptions became apparent of miRNAs not following this canonical biogenesis way. These exceptions include mirtrons, that are cleaved by the spliceosome independent of DROSHA^29, 30^, non-mirtron miRNAs e.g. miR-320 independent of DGCR8^31^, or miR-451, that is cleaved by Ago2 independent of DICER^32^. Therefore, it is difficult to maintain standard requirements that all “miRNAs” should meet, since the more stringent criteria would omit most non-canonical miRNAs, while weaker requirements would possibly lead to miss-annotations of other RNA species as “miRNAs”. Originally, miRNAs were identified by cloning and sequencing of cDNA libraries and detection of the endogenous miRNAs was confirmed by Northern blotting. Therefore, mainly miRNAs expressed at high levels or common to many tissues were identified. This scenario changed since the advent of next generation sequencing methods, where the number of identified potential miRNAs has risen dramatically^4^. This increase of potential miRNAs comes, however, with a significant drawback: many recently discovered miRNAs have been predicted based on a relatively low number of reads. The fewer reads for a miRNA candidate a library contains, the more unreliable is the exact prediction of the mature miRNA or the precursor sequence. Furthermore, since the candidate miRNA only obtains a handful of reads, we can assume a relatively low expression in the respective probe, which makes it difficult to validate endogenous levels of the candidate miRNA by Northern blotting. In fact, most miRNAs identified with NGS data still lack experimental validation by Northern blotting. It remains to be determined how many of the miRNAs identified using NGS sequences and computational predictions of a stem loop structure are really true miRNAs and not false-positives. False positive miRNAs may be short sequences, that stem from other RNAs or degradation products thereof, or from false annotated fragments in the genome with an artificial stem loop sequence predicted^4^. Several authors, including ourselves, expressed doubts about the authenticity of such novel miRNAs^4, 33–37^. By reanalysing data from mouse, Chiang *et al*. reported for around a third of the annotated mouse miRNAs a lack of convincing evidence that these precursors are able to produce authentic miRNAs^34^. Our own data clearly show a shift in sequence features of lately identified miRNAs in comparison to the first reported miRNAs (up to miRBase v7)^36^. Meng *et al*. reanalysed registered miRNAs in several plant species and found numerous instances with “miRNA” precursors that were predicted to have a malformed appearance with either missing 2 nt 3′ overhang or mature sequences inside the hairpin structure instead of on opposite arms^35^. There is also evidence of falsely annotated miRNA in humans. Hansen *et al*. performed a cross analysis of nearly 20 NGS data sets for human miRNAs and compared the results to the registered miRNAs of miRBase v16^37^. For a total of 102 precursors they found no read, 34 precursors were only supported by a single read, and for 95 precursors, read distribution showed signs of random degradation rather than a miRNA supporting distribution. Castellano *et al*. found several instances of small RNAs originating from other RNAs such as tRNA or rRNAs miss-annotated as miRNAs^38^. Aside from these falsely annotated miRNAs that represent no genuine miRNAs, discrepancies in sequences of known genuine miRNAs have been reported. Wang *et al*. compared miRNA reads from GEO data sets with the annotated miRNA sequences in miRBase version 14 and found that 19 of 174 *C. elegans* and 51 of 157 *D. melanogaster* miRNAs are miss-annotated in miRBase with either inconsistent 3′ or 5′ ends or miss-annotated guide and passenger strands^39^. In light of the questionable authenticity of a considerable portion of the miRNA registered in miRBase, we would recommend validation of a potential miRNA biomarker by Northern blotting to verify the miRNA nature of the marker before considering biological impact of the marker on cells based only on the assumption of a genuine miRNA.

In conclusion, we provide convincing evidence that RNA quality influences the miRNA expression profile, at least when measured using microarrays, and that RNA integrity and time to freezing should be carefully considered when collecting clinical samples. We also found evidence that at least part of the more recently identified miRNAs might indeed be falsely annotated miRNAs. For the identification of future biomarkers that may even serve as therapeutic targets in a clinical setting, we would strongly suggest to challenge the authenticity of miRNAs with an increased expression in the degraded tissues.

## Online Methods

### Tissue Samples

We obtained fresh cadavers of 6 male C57BL6 mice (Il1a knock-out strain) from the Department of Internal Medicine, Nephrology and Hypertension, Saarland University Medical Center. For determination of tissue specific degradation effects, heart, brain and liver of these animals were harvested immediately after death, divided into eight parts of about equal size and transferred in individual RNase free tubes. Tissue samples of three animals were stored at 4 °C and room temperature (18–20 °C), respectively. Tissue samples were transferred into RNAlater RNA Stabilization Reagent (Qiagen, Hilden, Germany) after storage for 0 h, 1 h, 3 h, 6 h, 14 h, 24 h, 48 h and 96 h at the respective temperature and stored at 4 °C for 24 h before freezing at −80 °C. In total, we collected 144 tissue samples, with three biological replicates for each time point at each temperature (Fig. 1a).

For determination of non-miRNA mediated background signals due to contamination of the sample with degraded DNA or RNAs, we obtained fresh liver samples of 3 mice and divided each liver in five parts of equal size. Three parts of each liver were transferred immediately into RNAlater RNA Stabilization Reagent (Qiagen, Hilden, Germany), i.e. non-degraded samples, the two remaining parts were left at RT for 96 h before transfer into RNAlater, i.e. degraded samples. For each experimental condition, we obtained three biological replicates (Fig. 1b).

### RNA isolation

For determination of tissue specific degradation effects, total RNA including miRNAs were isolated using miRNeasy Mini Kit (Qiagen, Hilden, Germany) according to the manufacturer’s manual. In detail, frozen tissue was homogenized in 700 μl QIAzol Lysis Reagent with using Tissue Lyser (Qiagen, Hilden, Germany) and incubated at room temperature for 5 min. After addition of 200 µl chloroform, samples were centrifuged at 12,000 rpm at 4 °C for phase separation. Aqueous phase was transferred into a new collection tube, mixed with 1.5 volumes 100% ethanol and applied to an RNeasy column. Column purification was performed according to the manufacturer protocol without the optional DNAse treatment, and RNA was eluted in 50 µl RNase free water.

For determination of background signals due to potential DNA contamination, non-degraded and degraded samples from three animals were isolated each with miRNeasy Kit either with or without optional on-column DNAse digestion. For determination of non-miRNA mediated background due to potential contamination with fragments of other RNAs during RNA degradation, total RNA was isolated from samples of three animals using RNeasy mini Kit (Qiagen, Hilden, Germany) with on column DNase digestion. We denominate this RNA as “RNA depleted of small RNAs”, being aware, that they are not 100% free of any miRNA or small RNA fractions, but the abundance of small RNAs in these samples is greatly reduced in comparison to the isolation procedure using the miRNeasy Kit. Intact RNA of these samples was aliquoted in 3 µg aliquots and treated with either 0 U, 0.027 U or 0.67 U RNase (Roche, Cat. No.11412000) for 30 min at 37 °C to obtain intact, partially degraded and fully degraded RNA, respectively. Afterwards, RNAse was inactivated with 700 µl Qiazol and RNA including any small fragments was reisolated using miRNeasy Mini Kit (Qiagen, Hilden, Germany) according to manufacturers protocol.

RNA quantity was assessed with Nanodrop (Thermo Fisher Scientific, Waltham, MA, USA), RNA integrity was determined with Agilent RNA 6000 Nano Kit using Bioanalyzer (Agilent Technologies, Santa Clara, CA, USA) for all samples. For determination of RNA degradation, RIN value was used.

### Microarray Profiling

Expression profile of miRNAs was assessed using SurePrint Mouse miRNA Microarray Kit, Release 21.0 and miRNA Complete Labeling and Hyb Kit (Agilent Technologies, Santa Clara, CA, USA) after manufacturer´s instructions as described elsewhere ^40^. Signals were retrieved using Agilent AGW Feature Extraction software (version 10.10.11). Background corrected values and detection flags for all miRNAs were extracted. Raw data has been deposited at GEO database (GSE93634). For determination of tissue specific degradation effects, we used 48 of the 144 collected samples, i.e. we selected liver, brain and heart tissue of the same individual at the 8 time points and two temperatures. For determination of non-miRNA mediated background signals due to possible contamination by residual DNA or RNA-degradation products, we used three biological replicates for each experimental condition, totalling 24 samples.

### Statistical analysis

Background corrected expression values were normalized using variance stabilizing normalization and log-transformed with freely available R software (v.2.14.2)^41^ (https://www.r-project.org/). All miRNAs detected in less than five samples were excluded, leaving 815 miRNAs for analysis of the tissue specific degradation effects and 791 miRNAs for determination of non-miRNA mediated background. Unsupervised hierarchical clustering using Euclidian distance and complete linkage has been applied.

P-values were adjusted using the Benjamini-Hochberg adjustment method^42^. We considered only miRNAs differentially expressed, that showed at least a 2-fold decreased or increased mean expression value and a p-value < 0.05 in WMW test.

## Electronic supplementary material

Supplementary Figures

Supplementary Table 1

Supplementary Table 2

Supplementary Table 3

Supplementary Table 4
