# Supplementary material for: Bias in recent miRBase annotations potentially associated with RNA quality issues
Source: Sci Rep. 2017 Jul 12;7:5162. doi: 10.1038/s41598-017-05070-0 (PMC5507985; doi:10.1038/s41598-017-05070-0)

Bias in recent miRBase annotations potentially associated with RNA quality issues

Nicole Ludwig[1], Meike Becker[1], Timo Schumann[2], Timo Speer[2], Tobias Fehlmann[3] Andreas Keller[3], Eckart Meese[1]

[1] Department of Human Genetics, Saarland University, Homburg, Germany
[2]Department of Internal Medicine, Nephrology and Hypertension, Saarland University Medical Center, Homburg, Germany.
[3] Chair for Clinical Bioinformatics, Saarland University, Saarbrücken, Germany

Supplementary Figures

Figure Legends:

Supplementary Figure S1: RNA Integrity during tissue degeneration. **a.** Representative Gel-like Images of RNA integrity in degrading tissues for the indicated conditions. **b.** Mean RIN values of isolated RNAs from heart, brain and liver tissues after storage for 0 to 96 hours at 4°C (left) and room temperature (right). Green, yellow and red shading indicates RIN values of high- medium and low quality RNA. Error bars for standard deviation were omitted for figure clarity. RIN values of individual samples can be found in Supplementary Table S1.

Supplementary Figure S2. Influence of degradation on miRNA expression profiles. Unsupervised hierarchical clustering using Eucledian distance and complete linkage of the samples depending on expression of the 50 miRNAs with highest variance. Blue color indicates high, red color indicates low expression. Samples are indicated on the X axis below the heatmap with each sample referring to the animal (first number), the tissue (li indicating liver, he for heart, br for brain), and the storage time in hours. The three tissues are also indicated above the heatmap by a color code with red for liver, green for heart and blue for brain. MiRNAs are indicated on the Y axis with the miRNA names on the right-hand side and the miRNAs cluster on the left-hand side.

Supplementary Figure S3: MiRNAs with highest positive and negative correlation with RNA integrity and storage time. **a.** MiRNA expression values as function of RIN for miR-467-3p (left) and miR-149-3p (right). **b.** Time course of expression values for miR-804 (left) and miR-652-5p (right) at 4°C. **c.** Time course of expression values for miR-6769b-5p (left) and miR-6366 (right) at room temperature. Pearson correlation coefficient (r) and significance (p) are given in each diagram.

Supplementary Figure S4: Expression of miRNAs in selected miRNA clusters. Box-plot of the median expression of miRNAs in clusters 4 (a), 5 (b), 6 (c) and 8 (d) of Fig. 5b. Box-plots of the median expression of miRNAs (Y axis) in liver tissues are shown for treatment/source of RNA as indicated above the box-plots (description is as in Fig. 6). The list of miRNAs belonging to each cluster can be found in Supplementary Table S3.

Supplementary Figure S5. Level of sequence homology to mRNAs in the eight miRNA clusters. Percentage of sequence homology of miRNAs in clusters 1 through 8 with coding sequence (red) or intron/UTR sequences (blue) of mouse mRNAs. Sequence homology is defined as minimum of 90% overlap with mature miRNA sequence with 1 mismatch allowed.

Supplementary Figure S1

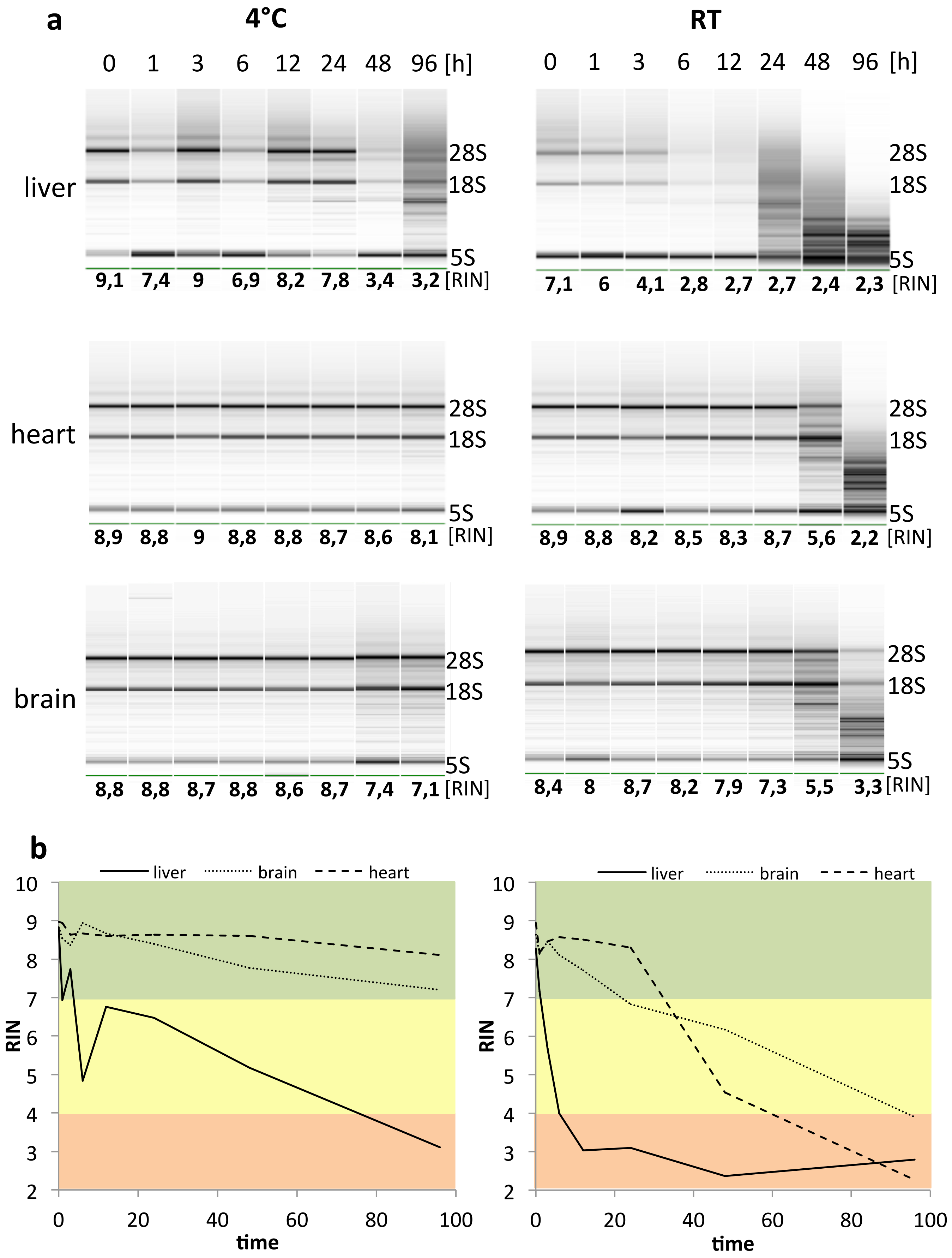

Supplementary Figure S2

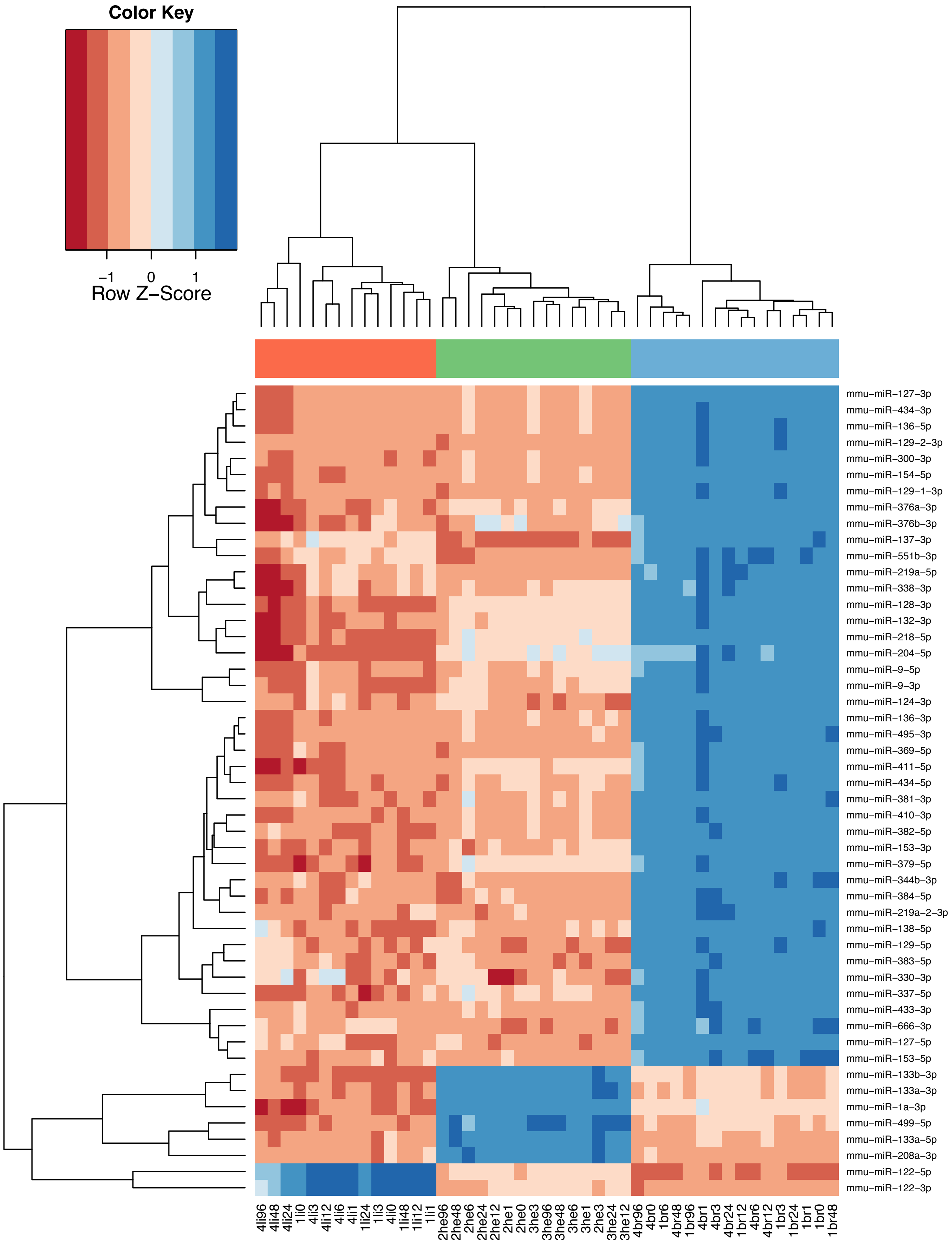

Supplementary Figure S3

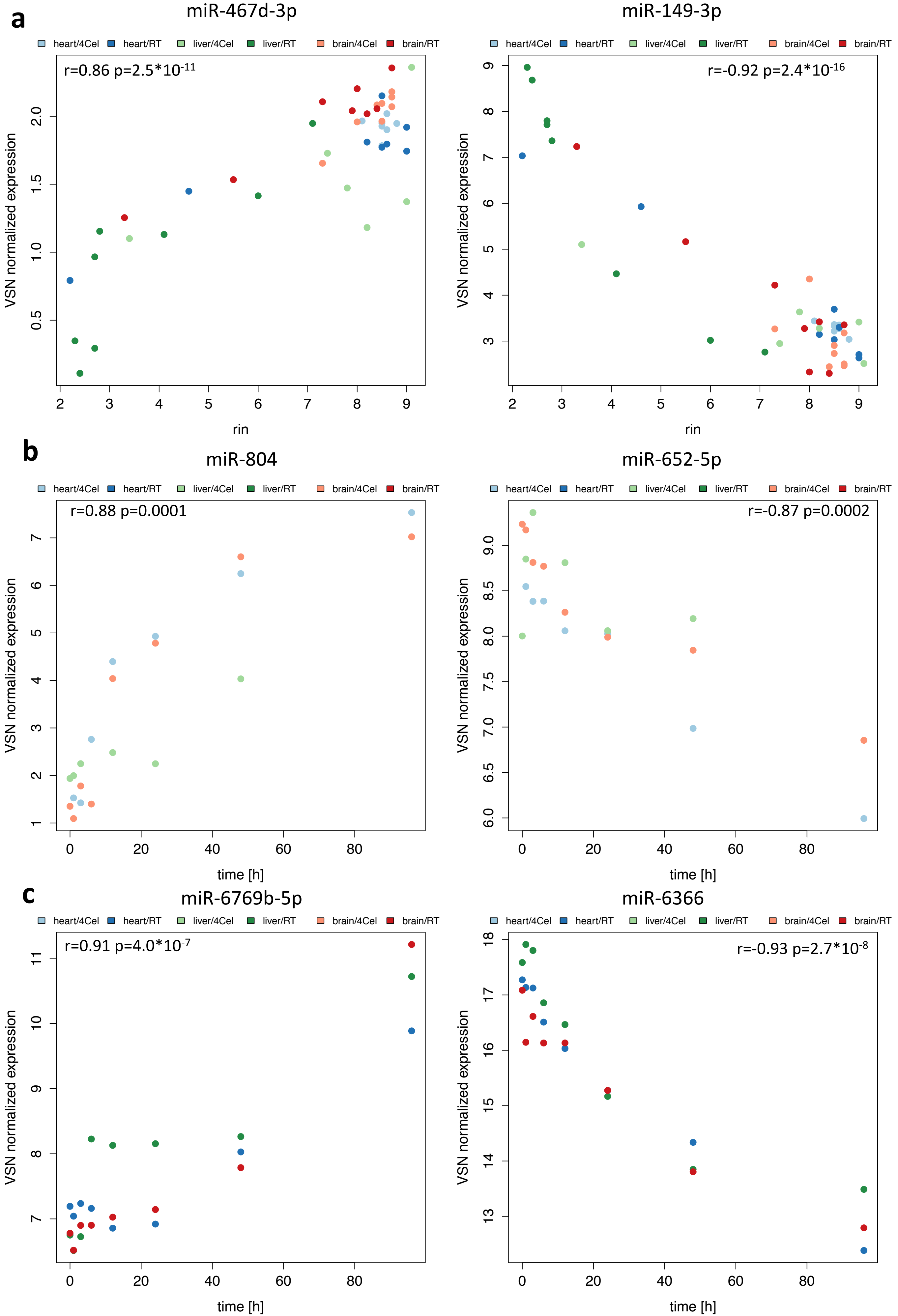

Supplementary Figure S4

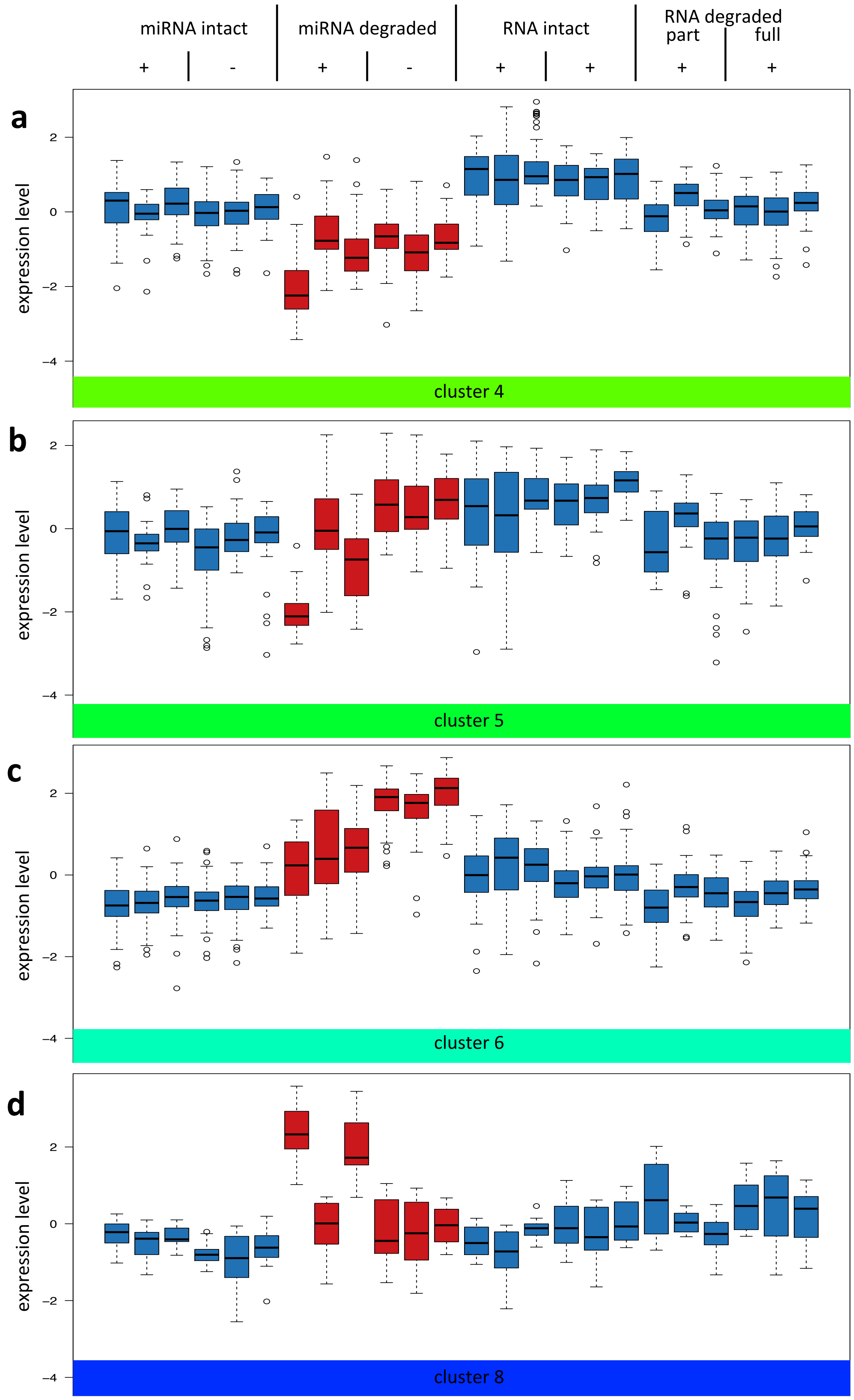

Supplementary Figure S5

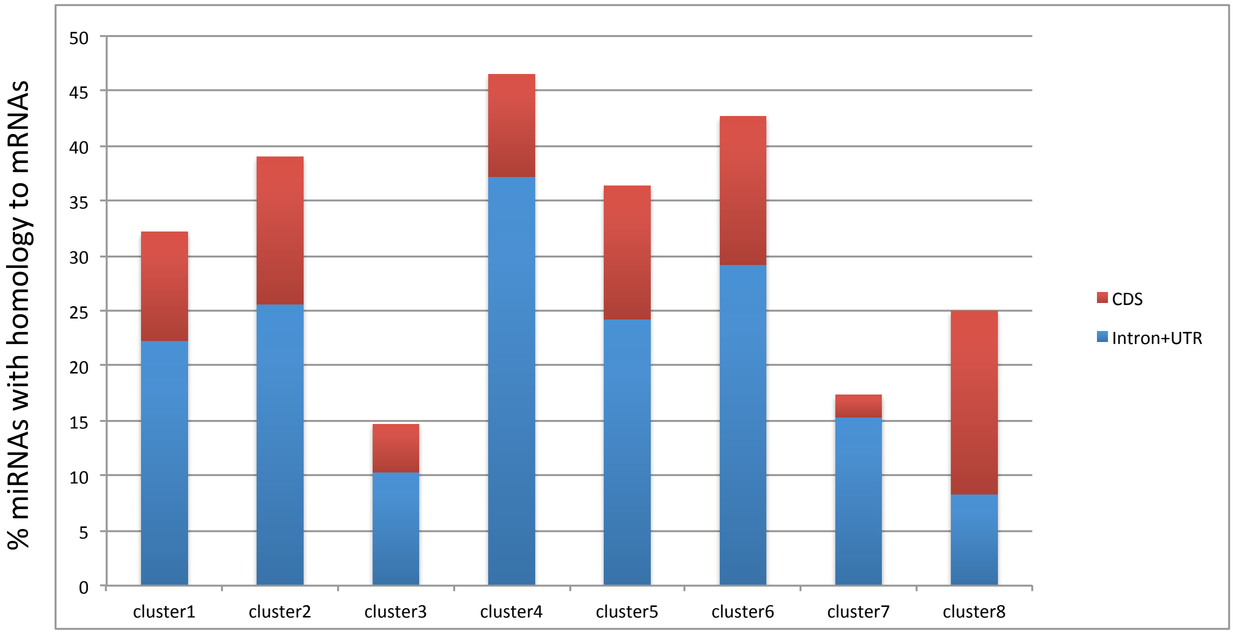

Supplement: Supplementary file 1 — Supplementary Figures [file 41598_2017_5070_MOESM1_ESM.pdf]
